# ncRNAs: an unexplored cellular defense mechanism in leprosy

**DOI:** 10.3389/fgene.2023.1295586

**Published:** 2023-12-04

**Authors:** Mayara Natália Santana-da-Silva, Camille Sena-dos-Santos, Miguel Ángel Cáceres-Durán, Felipe Gouvea de Souza, Angelica Rita Gobbo, Pablo Pinto, Claudio Guedes Salgado, Sidney Emanuel Batista dos Santos

**Affiliations:** ^1^ Laboratório de Genética Humana e Médica, Instituto de Ciências Biológicas (ICB), Universidade Federal do Pará (UFPA), Belém, Brazil; ^2^ Laboratório de Imunologia, Seção de Virologia (SAVIR), Instituto Evandro Chagas, Ananindeua, Brazil; ^3^ Laboratório de Dermato-Imunologia, Instituto de Ciências Biológicas (ICB), Universidade Federal do Pará (UFPA), Belém, Brazil

**Keywords:** leprosy, ncRNAs, miRNAs, *Mycobacterium leprae*, infection, immunity

## Abstract

Leprosy is an infectious disease primarily caused by the obligate intracellular parasite *Mycobacterium leprae*. Although it has been considered eradicated in many countries, leprosy continues to be a health issue in developing nations. Besides the social stigma associated with it, individuals affected by leprosy may experience nerve damage leading to physical disabilities if the disease is not properly treated or early diagnosed. Leprosy is recognized as a complex disease wherein socioenvironmental factors, immune response, and host genetics interact to contribute to its development. Recently, a new field of study called epigenetics has emerged, revealing that the immune response and other mechanisms related to infectious diseases can be influenced by noncoding RNAs. This review aims to summarize the significant advancements concerning non-coding RNAs in leprosy, discussing the key perspectives on this novel approach to comprehending the pathophysiology of the disease and identifying molecular markers. In our view, investigations on non-coding RNAs in leprosy hold promise and warrant increased attention from researches in this field.

## 1 Introduction

Leprosy (also known as Hansen’s disease) is a dermatoneurological disease that progresses to deformities and incapacities if not diagnosed and treated correctly (Britton and Lockwood, 2004; Bhat and Prakash, 2012). The causative agent of leprosy, *Mycobacterium leprae*, is known for a tropism for the upper respiratory tract, skin macrophages and Schwann cells (SCs) of peripheral nerves, conferring neurodermatological manifestations, including hypopigmented or erythematous cutaneous patches, sensorimotor loss and thickened peripheral nerves (Britton and Lockwood, 2004; Bhat and Prakash, 2012; Fava et al., 2019).

Leprosy is a disease that remains quite stigmatized, constituting a serious public health problem in countries like India, Brazil and Indonesia, where there is still a high prevalence rate (WHO, 2021a). It is important to emphasize that according to WHO data, during the year 2020 there was a decrease in the detection of new cases of the disease by about 37% compared to the previous year due to the pandemic of COVID-19 (WHO, 2021b). As a consequence of the pandemic, leprosy detection and treatment have been seriously affected due to staff shortages, suspension of activities in communities, delayed drug supply, etc. This disruption may result in an increase in cases and individuals with grade 2 disability (G2D) (WHO, 2021b; Paz et al., 2022).

The clinical manifestations of leprosy are presented as a spectrum, being intrinsically related to the host’s immune response against *M. leprae* (Fitness et al., 2002). Based on clinical, pathological, bacilloscopic and immunological criteria, leprosy can be classified as: tuberculoid (TT), borderline tuberculoid (BT), borderline borderline (BB), borderline lepromatous (BL) and lepromatous (LL) (Ridley and Jopling, 1966). Another type of classification, currently used by the World Health Organization, is based on the number of lesions: patients with up to five lesions are classified as paucibacillary (PB) and, patients with over five lesions, as multibacillary (MB) (WHO, 2019).

Importantly, it is still not entirely clear how immune response initiates in leprosy infection. We know that, at the TT pole, the infection is limited by a strong cell-mediated immunity, guaranteed by Th_1_ CD4^+^ cells, that secretes interleukin 2 (IL-2), and interferon (IFN)-γ that enhance macrophages and natural killers (NKs) microbicidal activities; therefore, in TT lesions, a granuloma formation and a low number of bacterial are observed (Modlin, 1994; Misra et al., 1995; Mi et al., 2020). As for LL lesions, the cell-mediated immunity is absent, giving place to Th2 antibody response, that produces IL-4 and IL-5 in abundant levels, which are known to activate B cells to switch antibodies (Mi et al., 2020). However, the humoral immunity against *M. leprae* is ineffective, allowing the bacilli to multiply in large number and disseminate the disease (Misra et al., 1995; Mi et al., 2020).

It is estimated that only a minority proportion of individuals exposed to *M. leprae* become infected (Nunzi and Massone, 2012; Alemu Belachew and Naafs, 2019). The explanation for such variability can be addressed to different reasons, including environmental factors, divergence in pathogen burden and human genetic susceptibility (Fava et al., 2019). In the global literature, there is evidence from a wide variety of studies supporting that host genes play an important role in susceptibility to leprosy in its different clinical forms, ranging from a classic twin study in the mid-twentieth century (Brown and Stone, 1958) to more recent Genome-Wide Association Studies (GWAS) (Wang et al., 2016; Gzara et al., 2020), which may elucidate leprosy pathology with the investigation of different immune-related genes (Fitness et al., 2002; Fava et al., 2019; Mi et al., 2020).

Currently, it is known that the immune response and other related mechanisms may be influenced not only by genetic factors but also by epigenetic regulation, which includes the activity of non-coding RNAs (ncRNAs) (Cavalli and Heard, 2019). *M. leprae* alter host cell functionality to their own advantage to promote survival and generate a suitable environment for replication within the host cell by modifying host epigenome (Niller and Minarovits, 2016; Zhang and Cao, 2019). In the last few years, it has been demonstrated that ncRNAs are broadly involved in the activation or suppression of the expression of distinct gene sets related to leprosy phenotype, which directed to novel knowledge on the role of ncRNAs in immunity generation and disease progression, although much remains to be discovered. Here, we review the recent advances in understanding ncRNAs-mediated regulation on leprosy physiopathology and we discuss their importance as potential biomarkers for this disease.

## 2 Epigenetics

Epigenetics could be defined as the study of molecules and mechanisms capable of modifying regulation and gene expression, without altering the genomic sequence (Cavalli and Heard, 2019). The main mechanisms related to epigenetic regulation are DNA methylation (Moore et al., 2013), histone modifications (Stillman, 2018) and, as previously mentioned, ncRNAs (Chuang and Jones, 2007; Han and He, 2016). These host mechanisms can become excellent tools for pathogens, providing persistent infections by the downregulation of the immune response through bacterial factors capable of altering various cell signaling pathways (Bierne et al., 2012; O’Connell et al., 2012; Syn et al., 2016).

The ncRNAs are RNA molecules that may have structural, functional and regulatory roles, representing about 80% of the genome (ENCODE Project Consortium, 2012). Non-coding RNAs have the capacity to interact with DNA, RNA, and proteins, and they serve multiple functions. These functions include acting as cis-acting silencers and trans-acting mediators at specific transcriptional loci, as well as participating in post-transcriptional processes, nuclear organization, RNA processing, and the suppression of transposons through sequence complementarity (ENCODE Project Consortium, 2012; Hombach et al., 2016; López-Jiménez and Andrés-León, 2021).

**FIGURE 1:**
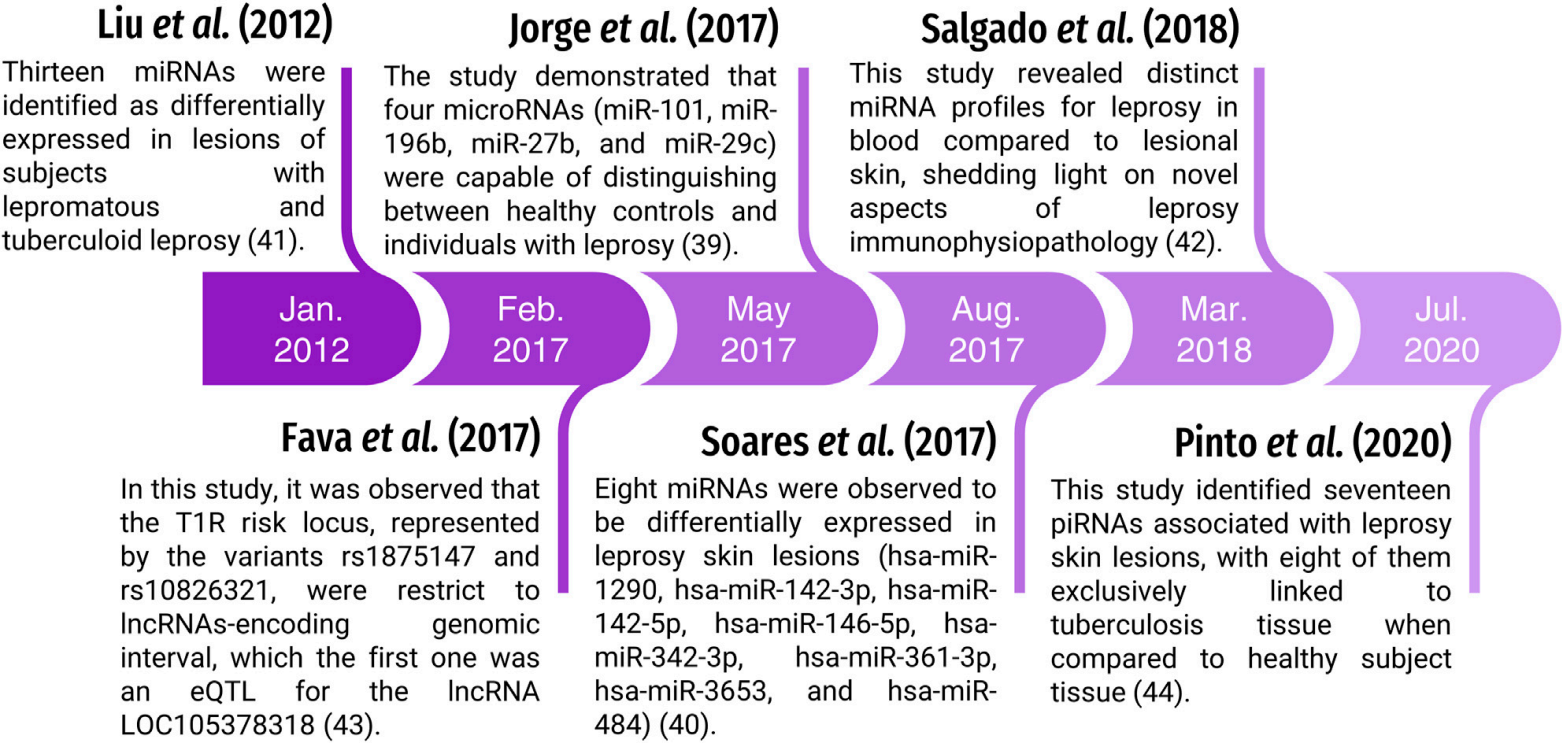
Timeline of studies involving ncRNAs and leprosy.

Based on size, biogenesis and structure, ncRNAs are grouped into two major groups: long noncoding RNAs (lncRNAs >200 nt) and small noncoding RNAs (sncRNAs <200 nt) (Charles Richard and Eichhorn, 2018; Bhatti et al., 2021). Currently, there is increasing evidence that deregulation in the transcription and maturation of ncRNAs, incorrect interaction with target mRNAs and mutations in the processing mechanism can increase the risk of neurological, cardiovascular diseases and tumorigenesis (Calin et al., 2004; KAZEMZADEH et al., 2015; Anastasiadou et al., 2018; Sallam et al., 2018; Yan et al., 2018; Grillone et al., 2020). However, there are still few studies on the association of ncRNAs with infectious diseases, such as microRNAs (miRNAs) (Liu et al., 2012a; Jorge et al., 2017; Soares et al., 2017; Salgado et al., 2018), lncRNAs (Fava et al., 2017) and piwi-interacting RNAs (piRNAs) (Pinto et al., 2020) in leprosy (Figure 1) (Cokarić Brdovčak et al., 2018; Schulte et al., 2019; Fathizadeh et al., 2020; Pinto et al., 2020). To date, no original articles have been published addressing the role of ncRNAs in leprosy. Therefore, in the following sessions, we will focus on these classes of ncRNAs and their relation to leprosy. Table 1 presents their main characteristics.

**TABLE 1:** Characteristics of the main classes of ncRNAs.

Class	Length (nt)	Functions	References
LncRNAs	>200	Regulation of gene expression in the transcriptional and post-transcriptional levels, chromatin remodeling, miRNA sponge	Quinn and Chang (2016), Yao et al. (2019)
miRNAs	19–24	RNA and gene silencing, gene expression activation, gene modulation at the post-transcriptional level	Ramchandran et al. (2017), Yang and Ge (2018), Lukasik and Zielenkiewicz (2019)
piRNAs	26–31	Epigenetic and post-transcriptional gene silencing	Iwasaki et al. (2015), Ozata et al. (2019), Pinto et al. (2020)
circRNAs	>200	Modulating transcription and splicing, miRNA sponge and suppress transcription and gene silencing	(Huang et al., 2020; Li et al., 2018a)

### 2.1 LncRNAs

Long non-coding RNAs (lncRNAs) are functionally heterogeneous molecules that present a length of at least 200 nt, a lack of protein-coding potential, usually a poly-A tail and that may be spliced, similar to mRNAs (Hombach et al., 2016). They are classified according to the relative location to protein-coding genes as: sense lncRNA, antisense lncRNA, bidirectional lncRNA, intron lncRNA, intergenic lncRNA and enhancer lncRNA (Chi et al., 2019).

LncRNAs have been associated with several functions and represent the largest class of ncRNAs. Contrary to short ncRNAs, which are generally attributed to gene regulation, the mechanistic role of lncRNAs is highly diverse, increasing their functional complexity (Charles Richard and Eichhorn, 2018). At the epigenetic level, lncRNAs can regulate DNA methylation, alter methylation, acetylation, or ubiquitination of histones and reconstruct the chromatin or alter its conformation (Zhang T. et al., 2019). It has been demonstrated that lncRNAs play important roles in stem cell maintenance and differentiation, X-chromosome inactivation, imprinting, maintenance of nuclear architecture, cell autophagy, cell proliferation, apoptosis and embryonic development (Hombach et al., 2016; Qian et al., 2019).

In the immune system, lncRNAs exhibit dynamic expression in cell type, developmental stage, and context-specific manners to coordinate several aspects of immune function (Atianand and Fitzgerald, 2014). The majority of lncRNAs that are associated with infection diseases have been shown to dysregulate expression in different tissues and cells. Several studies have found differentially expressed host lncRNAs in various bacterial infections such as *Escherichia coli*, *Salmonella*, *Campylobacter concisus* and *Mycobacterium tuberculosis* (Li et al., 2018a; Chi et al., 2019; Ozata et al., 2019; Huang et al., 2020), indicating that lncRNAs can be used as molecular markers of infection associated with pathogenic bacteria.

To date, the only study that has directly related lncRNAs and leprosy was done by Fava and collaborators (Fava et al., 2017). In this genome-wide association study, the authors have identified a lncRNA as a potential risk factor for the development of pathological inflammatory responses in leprosy. Specifically, two risk variants associated with leprosy type-1 reactions (T1R), namely, rs1875147 and rs10826321, are found to be linked to two distinct isoforms of a novel lncRNA. One of these isoforms is encoded by the *ENSG00000235140* gene, also known as *RP11-135D11.2*, while the other is encoded by the uncharacterized *LOC105378318* gene. It’s noteworthy that these two genetic variants are situated at positions 6.5 kb and 8.7 kb, respectively, upstream of the transcription start site of the *ENSG00000235140* gene. These T1R events manifest as pathological inflammatory responses affecting a specific subgroup of leprosy patients, often leading to peripheral nerve damage. These episodic occurrences significantly contribute to immune-mediated tissue damage in the host and, consequently, play a pivotal role in the development of disability in leprosy (Fava et al., 2017).

Another study, using learning machine to predict leprosy progression amongst household contacts of leprosy patients, identified a group of genes among which three lncRNAs were found (*ENSG00000283633*, *ENSG00000266538* and *ENSG00000279227*). However, these lncRNAs, along with other pseudogenes were not included for validation of the RNA-Seq signature due the lack of commercially available probes for RTqPCR (Tió-Coma et al., 2021).

Currently, research on lncRNAs in leprosy and other infectious immunity is just beginning when compared to studies on other ncRNAs, such as miRNAs, in this field. One of the major challenges in identifying lncRNAs relates to difficulty in discriminating their pleiotropic functions, as well as understanding the mechanisms by which they interact with other molecules, such as proteins, miRNAs, mRNAs, and circRNAs. Similarly, the interaction of lncRNAs with the immune system has not yet been fully elucidated, being needed more experimental and clinical studies to offer novel approaches for better diagnosis and therapy in the future.

### 2.2 miRNAs

Among the variety of ncRNAs, the most frequently studied are miRNAs. Discovered as non-coding and post-transcriptional regulators in eukaryotes in the early 1990s (Lee et al., 1993), miRNAs are small, endogenous, stable, and highly conserved among species. MiRNAs are predicted to control the activity of approximately 30% of all protein-coding genes in the human genome (Friedman et al., 2009), and have been shown to participate in the regulation of thousands of genes (Schulte et al., 2019). They are among the most important regulatory molecules of an organism and participate in a variety of biological processes that include the modulation of the immune response during infections (Felekkis et al., 2010; Zhou et al., 2010; Singh RP. et al., 2013; Bhaskaran and Mohan, 2014; Lukasik and Zielenkiewicz, 2019).

However, studies that associate the expression of miRNAs in infectious diseases are still insufficient, especially related to diseases caused by mycobacteria (Singh RP. et al., 2013; Yang and Ge, 2018; Abu-Izneid et al., 2020). It is known that these microorganisms have various routes of infection and can cause diverse immune response based on the cells that are likely infected (Agarwal et al., 2019) For example, the main cell types directly infected by the mycobacteria are macrophages, which are crucial modulators of innate and adaptive immune responses, leading to different immune responses by deregulation of host miRNAs (Singh PK. et al., 2013; Bettencourt et al., 2016).

**FIGURE 2:**
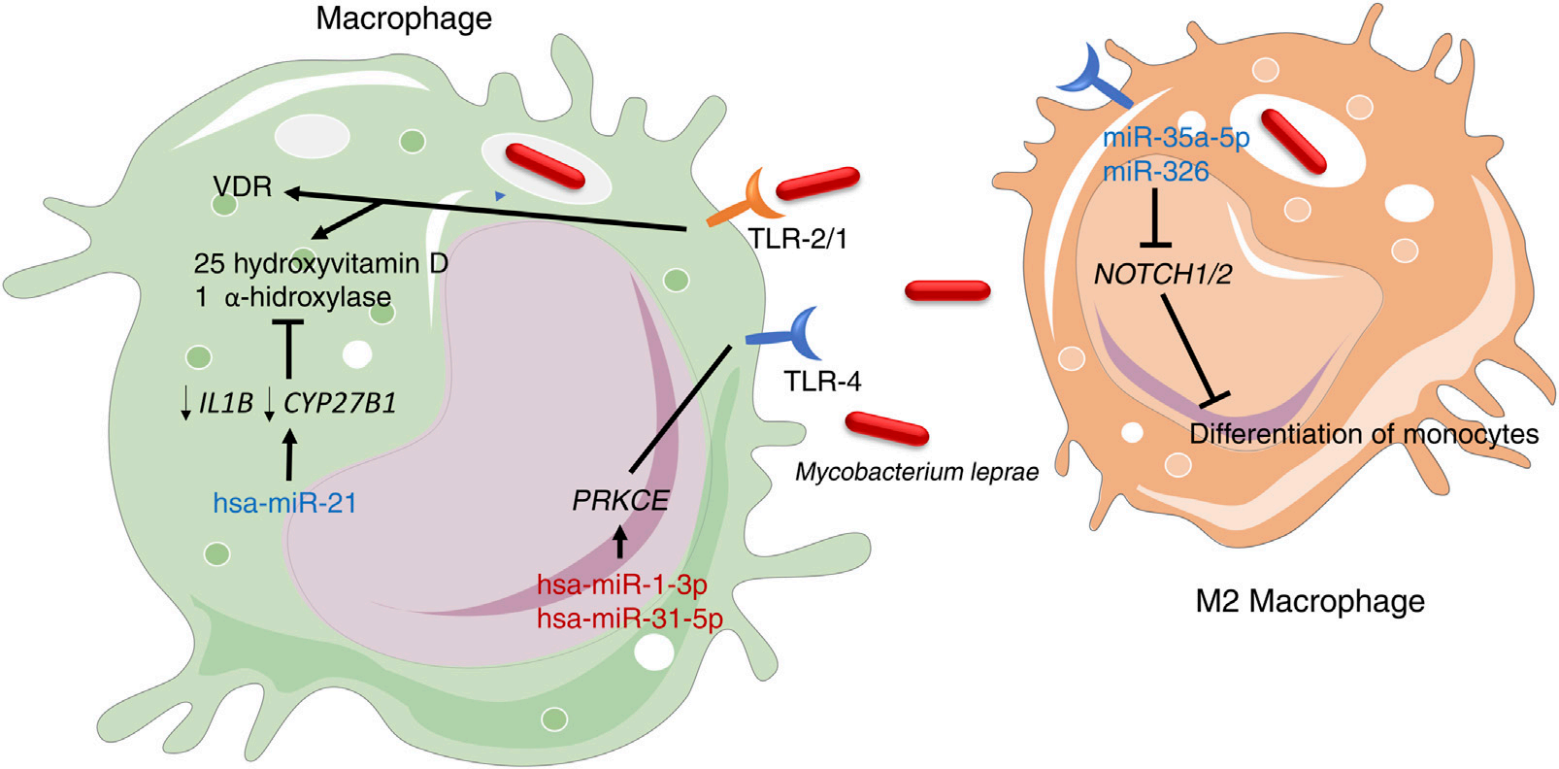
TLR-4 and TLR-1/2 are crucial receptors involved in the recognition of *M. leprae* in macrophages. miRNAs highlighted in blue indicate high expression, while miRNAs in red indicate low expression.

Macrophages act in the front line of host defense and are a major target of these pathogens (Zhou et al., 2010; Singh RP. et al., 2013). In leprosy, the infection of macrophages results in the recognition by TLR-4 (Polycarpou et al., 2013) and TLR-2/1 (Krutzik et al., 2003), and subsequent direction of adaptive immune responses. Two miRNAs that target *PRKCE* gene (hsa-miR-1-3p and hsa-miR-31-5p), which in turn is involved in TLR-4 signaling in mycobacterial infections (Hestvik et al., 2005), showed low expression in leprosy tissues of both TT and LL poles through miRNA sequencing (Salgado et al., 2018). On the other hand, Liu et al. (2012b), using microarray analysis, demonstrated that in LL patients, the vitamin D-dependent antimicrobial effects of TLR-2/1 are directly inhibited by the overexpression of hsa-miR-21. This miRNA negatively regulates the expression of *IL1B* and *CYP27B1* genes, activates downstream of TLR-2/1 signaling, and plays a role in the induction of vitamin D immunomodulatory activities. This results in an indirect increase in the production of IL-10, subsequently leading to the inhibition of antimicrobial peptides CAMP and DEFB4A. (Liu et al., 2012b) (Figure 2).

The persistence of *M. leprae* infection depends on the type of the host immune response and macrophages present in the skin lesions. Macrophages have high plasticity and can polarize to different phenotypes, including M1 (pro-inflammatory profile) and M2 (anti-inflammatory profile), with mostly homeostatic and phagocytic functions (Kibbie et al., 2016). In the skin lesions of LL patients, miR-34a-5p and miR-326 were upregulated, causing the inhibition of *NOTCH1/2* expression and preventing the differentiation of monocytes to M1 macrophages (Salgado et al., 2018). Thus, the populations of M2 macrophages were found to be predominant in LL lesions, indicating that the types of macrophages present in lesions are associated with clinical spectrum of leprosy.

To initiate the adaptive response, the interaction between adhesion molecule 1 (CADM1) expressed on the surface of dendritic cells and the molecule associated with MHC class I restricted T cells (CRTAM) coordinates the immunological synapse required for T cell receptor (TCR) activation and polarization in subpopulations of CD4^+^ T cells (Yeh et al., 2008; Giangreco et al., 2012). MiRNAs can control the bridge between innate and adaptive immune signaling mechanisms and regulatory networks that determine which subpopulations of Th cells will be activated during leprosy (Baumjohann and Ansel, 2013; Kumar et al., 2013). The miR-15a-5 and miR-16-5p, that control the interaction between *CADM1* and *CRTAM* have been shown to be downregulated in all groups of leprosy patients (Salgado et al., 2018). However, the downregulation of miR-181a is associated with the progression of leprosy to BL and LL forms (Kumar et al., 2011). This miRNA is particularly important for TCR signaling for regulating SHP2, a phosphatase that reduces TCR expression and, as a consequence, SHP2 overexpression compromises TCR signaling and specificity of T cells against *M. leprae* (Kumar et al., 2011).

The regulatory network of miRNAs that operate in differentiation for TCD4+ Th1, TCD4+ Th2, and TCD4+ Th17 cells are still far from being fully comprehended. When overexpressed, miR-125b regulates genes involved in the differentiation of naive TCD4+ cells (e.g., *IFNG*, *IL2RB*, *IL10RA*, and *PRDM1*); whereas it is downregulated in leprosy patients, it does not seem to prevent the differentiation of TCD4+ in the subpopulations of Th cells (Rossi et al., 2011; Salgado et al., 2018). It has also been previously demonstrated *in vitro* that the overexpression of miR-155 in TCD4+ cells promote differentiation into Th1 cells, while the its downregulation appears to activate Th2 cells (Banerjee et al., 2010), but, in LL patients, miR-155 was upregulated (Salgado et al., 2018). In a previous study, miR-155 was associated with the repression of SHIP1 and the transcriptional repressor BTB and CNC homology 1 (Bach1), which results in increased activation of Akt (serine/threonine protein kinase B) and favors the survival of mycobacteria inside cells (Kumar et al., 2012; Rothchild et al., 2016). However, these results are still quite controversial, since studies show that miR-155 is also able to promote differentiation into Th17 cells through regulation of the *JARID2* gene (Escobar et al., 2014; Xu et al., 2017; Zhu et al., 2020).

The Th17 subset is usually found in patients with unstable leprosy, where there is absence of Th1 and Th2 polarization, and is also associated with T1R leprosy reactions (Nath et al., 2015; Saini et al., 2016). Similar to miR-155, the miRNA hsa-miR-326, which is reported to be an important inductor of differentiation of Th17 cells (Du et al., 2009; Karimi et al., 2020) was also found upregulated in LL (Salgado et al., 2018). On the other hand, some studies report the importance of miR-326 as a regulator of apoptosis in neuronal cells, able to inhibit JNK and MAPK signaling pathways when overexpressed (Zhang Y. et al., 2019; He et al., 2020). From an immunological point of view, apoptosis is a form of programmed cell death (PCD) that functions as a defense against infections, involving in the death of infected macrophages (Ottenhoff, 2012). Modulation of apoptosis can influence the course of infection, allowing intracellular pathogens to survive, and may be an important mechanism in the development of the various clinical forms of leprosy, as they are influenced by the bacillary load (Brito de Souza et al., 2010; Galluzzi et al., 2017).

**FIGURE 3:**
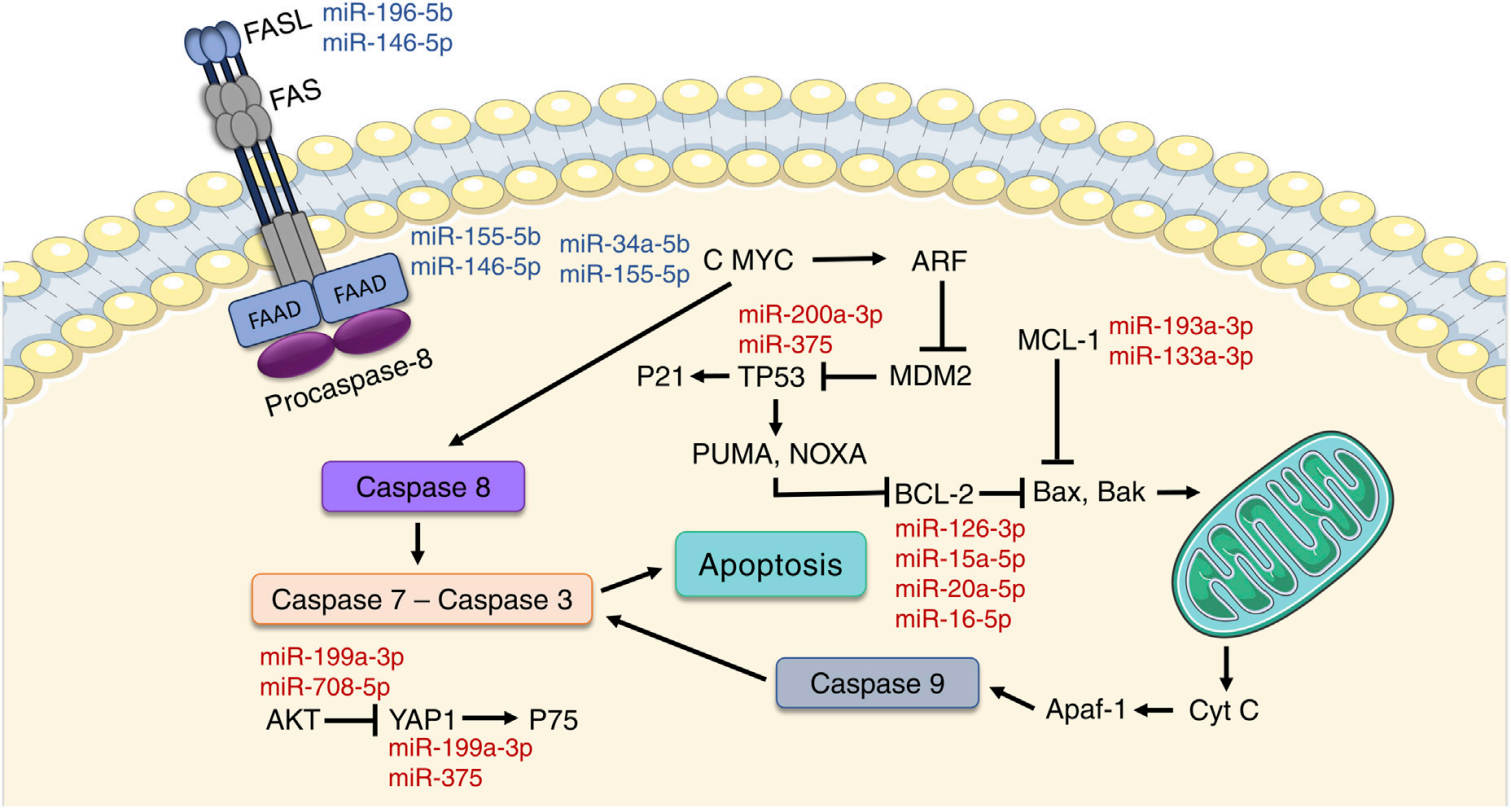
MicroRNAs regulates apoptosis-associated genes in leprosy. MicroRNAs are able to modulate both extrinsic and intrinsic apoptosis pathways during the infection process. The miRNAs that regulate the expression of genes associated with the intrinsic apoptotic pathway (*FASL*, *FAS*, *FADD*, and *CASP8*) are upregulated, while the miRNAs that control intrinsic signaling, highlighting the antiapoptotic gene *BCL-2*, are downregulated, demonstrating the anti-apoptotic profile of cells infected by *M. leprae*. miRNAs highlighted in blue indicate high expression, while miRNAs in red indicate low expression.

Several miRNAs that target Caspase-8 (CASP-8) inductor are upregulated in the two leprosy extreme poles. Apoptosis-related genes like *MYC* (miR-34a-5p and miR-155-5p), *FAS* (miR-196b-5p and miR-146a-5p) and *FADD* (miR-155-5p and miR-146a-5p) are upregulated only in LL patients, while miR-126-3p, miR-15a-5p, miR-20a-5p and miR-16-5p, that target *BCL-2*, an antiapoptotic gene, and miR-193a-3p and miR-133a-3p, that target *MCL1*, a member of BCL-2 family, were downregulated (Salgado et al., 2018). In LL lesions, miRNAs that target the pro-apoptotic gene *YAP1* (miR-200a-3p and miR-375) and miRNAs that target *AKT1* (miR-199a-3p and miR-708-5p) were all downregulated. *AKT1* is a known suppressor of the apoptotic machinery, including *YAP1* and *FOX03*. *TP53* also presented its miRNAs (IAmiR-200a-3p and miR-375) downregulated in LL lesions, but it has its functions blocked through activation of RBL1/2, that are activated after inhibition of *FOX03*, besides, *AKT1* also stimulates *MDM4*, a regulator of *TP53* (Figure 3). Therefore, *M. leprae* can impact macrophages miRNAs expression levels, thus altering apoptosis to save itself from intracellular death (Salgado et al., 2018).

In addition to understanding about the immunopathophysiology of leprosy, some studies have also sought biomarker miRNAs that are accessible and reliable. Jorge et al. (2017) studied sets of miRNAs differentially expressed by low-density array in skin lesions of LL and TT patients, with the expression levels of seven miRNAs (miR-125b, miR-196b, miR-27b, miR-29c, miR-425-5p, miR-502-3p, and miR-92a), and among these four miRNAs were selected (miR-101, miR-196b, miR-27b, and miR-29c) from evaluation by their ability to distinguish between group of patients and control in the first stage, and group of LL and TT in a second stage. The combination of miRNAs miR-101, miR-196b, miR-27b and miR-29c show 80% sensitivity and 91% specificity (AUC 87%) in discriminating patients with leprosy, and was also able to discriminate between TT and LL with 83% sensitivity and 80% specificity (AUC 83%).

In another study carried out by Soares and collaborators (Soares et al., 2017), comparing clinical forms (TT, BT, BB, BL, and LL) and a control group, twenty miRNAs were found differentially expressed, while in polar forms (TT and LL) only one miRNA was identified (hsa-miR181a). The hsa-miR181a is upregulated across the spectrum of leprosy and reaction forms, with greater expression of the polar forms TT and LL, which may indicate importance in the pathophysiological process of the disease (Soares et al., 2017). It is known that hsa-miR-181a is capable of regulating the sensitivity of T cells, allowing mature T cells to recognize antagonists as agonists (Li et al., 2007; Amado et al., 2020). The higher expression of hsa-miR-181a is correlated with greater sensitivity of T cells in immature T cells, suggesting that this miRNA acts as a rheostat of intrinsic sensitivity to the antigen (Li et al., 2007; Soares et al., 2017). Of the sixty-four miRNAs, eight were validated, seven of which were upregulated (hsa-miR-142-3p, hsa-miR-142-5p, hsa-miR-146b-5p, hsa-miR-342-3p, hsa-miR-361 -3p, hsa-miR-3653, and hsa-miR-484) and one were downregulated (hsa-miR-1290) (Soares et al., 2017).

In other study, da Silva MNS et al. (2022) found association of twenty-five genetic variants in miRNAs and miRNA machinery-related genes (*DROSHA* and *AGO1*) with leprosy susceptibility in a population from the Amazon region. In the association analysis between leprosy patients and healthy individuals, they found six significant markers for the risk of developing leprosy in the population studied: rs2505901 (pre-mir938), rs639174 (*DROSHA*), rs636832 (*AGO1*), rs10739971 (pri-let -7a1), rs12904 (miR200C) and rs10035440 (*DROSHA*).

Comparing leprosy patients grouped according to the PB form with the control group, they found a significant association in SNPs rs2505901 (pre-miR938), rs10739971 (pri-let-7a1), rs12904 (miR200C) and rs2168518 (miR4513) (da Silva MNS et al., 2022). The marker rs2505901 (pre-miR938) was associated with a decreased risk of PB leprosy in recessive and dominant models and changes in miR938 biogenesis and stability, in addiction to be associated with regulatory pathways related to cell survival and apoptosis (Nath et al., 2015; Zhu et al., 2020). On the other hand, SNPs rs10739971 (pri-let-7a1) and rs12904 (miR200C) were associated with increased risk of PB in a dominant model and rs2168518 (miR4513) in recessive model (da Silva MNS et al., 2022).

Comparing genotypes of patients grouped according to MB clinical form with the control group, SNPs rs639174 (*DROSHA*), rs636832 (*AGO1*) and rs4143815 (miR570) were associated with a reduced risk of MB leprosy using a dominant model (da Silva MNS et al., 2022). The rs639174 variant (*DROSHA*) and the rs4143815 variant (miR570) is an intronic SNP with a recognized role in transcriptional regulation and are involved in the regulation of the inflammatory response, respectively, and in this study this variants was associated with protection against leprosy *per se* and MB (Du et al., 2009; Saini et al., 2016; Zhu et al., 2020). On the other hand, SNP rs10035440 (*DROSHA*), which also plays an important role in the splicing and transcriptional regulation of the *DROSHA* gene, was associated with increased disease risk in a dominant model (Saini et al., 2016; Zhu et al., 2020).

In a genotype comparison analysis between PB and MB patients, only SNP rs10739971 (pri-let-7a1) showed an association with the development of leprosy *per se* and the PB form, with an association between groups of patients (PB versus MB), with increased risk to the PB form (106). While the rs2910164 SNP (miR146A) was associated with a decreased risk of leprosy in the PB form in a recessive model and consequent susceptibility to risk of MB leprosy (da Silva MNS et al., 2022).

Among ncRNAs, miRNAs are the most studied in leprosy, with studies aiming at basic knowledge of how these molecules act in the immune response and pathophysiology of the disease, aiming at new therapeutic targets aimed at the interaction between *bacillus*/host and accessible biomarkers. However, many of the miRNAs found require validation and functional analysis to assess their roles in the pathogenesis of leprosy.

### 2.3 piRNAs

PIWI-interacting RNAs (piRNAs) represent the most abundant and diverse group of sncRNAs (Ku and Lin, 2014), with more than 30,000 piRNAs molecules identified in the human genome (Chalbatani et al., 2019). Several genomic loci, defined as piRNA clusters, can transcribe pri-piRNA sequences (Brennecke et al., 2007), that unlike miRNA and siRNA, are processed through Dicer-independent mechanisms, to mature piRNAs (Vagin et al., 2006). Structurally, piRNAs are single-stranded molecules, typically 24–32 nucleotides in length, characterized by 2′-O-methyl-modified 3′ termini. These sncRNAs interact with proteins of the PIWI family, a sub-group of the AGO proteins, forming the piRNA-induced silencing complex (piRISC) (Siomi et al., 2011).

The involvement of piRNAs in the innate and adaptative immune response is not a well-established concept as for miRNAs, and few studies with controversial results have pointed out a possible relationship between the expression of piRNAs and the response to viral infections (Petit et al., 2016). Despite this, the pioneer work by Pinto et al. (2020) focused on the global changes in the piRNA expression profile (piRNome) of leprosy skin lesions, and detected 337 piRNAs in human skin, of which five piRNAs (piR-hsa-28634, piR-hsa-1580, piR-hsa-27007, piR-hsa- 21131, piR-hsa-12454) were differentially expressed (DE) in leprosy tissues when compared with healthy tissues (HS) (TT + LL vs. HS).

Other piRNAs were exclusively DE to the different leprosy poles: eight DE piRNAs (piR-hsa-23327, piR-hsa-23655, piR-hsa-2153, piR-hsa-12790, piR-hsa-31280, piR-hsa-28394, piR-hsa-27283, piR-hsa-23289) were found only in TT and three DE piRNAs (piR-hsa-23919, piR-hsa-26131, piR-hsa-15215) were found only in LL leprosy (Pinto et al., 2020). This indicates that piRNA expression profiles can distinguish leprosy tissue from a non-leprosy tissue, even more, can distinguish leprosy between its TT and LL poles, therefore, these piRNAs are attractive biomarkers for leprosy.

Under normal physiological conditions, the piRNAs are produced at stable levels (Story et al., 2019), however, in leprosy skin lesions all but one of DE piRNAs (piR-hsa-27283) are downregulated, emphasizing epigenetic alterations in leprosy (Pinto et al., 2020). Considering that, piR-hsa-27283, was the only piRNA upregulated, it may be useful as a risk biomarker of leprosy, more specifically, for TT patients, since it was DE for this pole, and as leprosy poles have different immunological and molecular features, it is suggested that piR-hsa-27283 functions can have a significant impact on progression to TT leprosy. On the other hand, its role in leprosy is unclear, as it may has multiple gene targets, thus making its analysis difficult (Pinto et al., 2020).

To better clarify the functional and mechanistic features of piRNAs in leprosy context, the authors built a piRNA-gene regulatory network, based on DE piRNAs putative target genes, which revealed that, in general, DE piRNAs regulate genes involved in leprosy-related processes, such as, programmed cell death (i.e., apoptosis, autophagic cell death), immune response, neural and epidermal regeneration (Pinto et al., 2020).

Lines of evidences suggest that immunological response generated during *M. leprae*, induces the apoptosis of infected cells, mainly by the secretion of pro-apoptotic cytokines, such as TNF and IFN, which are markedly dominant in TT lesions (Jin et al., 2018). However, synergistically with miRNAs, the piRNAs regulate anti-apoptotic pathways, via CARF activation, an inhibitor of caspase-dependent apoptosis, allowing a favorable condition to bacterial survival (Pinto et al., 2020).

An important question is if piRNAs are downregulated in leprosy biopsies and considering that SCs of peripheral nerves possess genomic plasticity and high regenerate capacity guaranteed by *SOX10* and *ERBB* gene family, why in leprosy the regrowth of axons and SCs is inhibited? (Tapinos et al., 2006; Hess and Rambukkana, 2019). Recently, Masaki et al. (2013) demonstrated that the leprosy bacterium hijacks this regenerative property and turn-off myelination-associated genes by DNA methylation of *SOX10* promotor region, a regulator of SCs differentiation and myelination, thus, the cells remain in an undifferentiated stage and become capable to migrate to other tissues, which facilitated the spread of *M. leprae* to other niches, such as skeletal and smooth muscle, and also contribute to granuloma formation that subsequently release of *M. leprae*-laden macrophages.

Another important mechanism for SCs regeneration is the recruitment of pro-regenerative macrophages. The pro-regenerative macrophages function to clear axonal and myelin debris, persisting in nerve microenvironment to guide remyelination and SC differentiation after nerve injury, which mechanism are regulated by growth arrest specific 6 (GAS6) and IL-6 (Stratton et al., 2018). It is proposed that the downregulation of piRNAs (piR-hsa-12454, piR-hsa-1580, piR-hsa-2153, piR-hsa-23289) that target IL6R to culminate in the expression of this receptor in pro-regenerative macrophages and active the IL-6, a well-known neuropathic biomarker in leprosy patients (Pinto et al., 2020). The IL-6 in turn, stimulates pro-regenerative macrophages to produce GAS6 that promotes better SCs remyelination within the injured nerve (Stratton et al., 2018).

Unfortunately, the work of Pinto and collaborators (Pinto et al., 2020) is the only one study available in the specialized literature that aimed to understand the involvement of piRNAs pathway in leprosy. Clearly, there is still a gap in this regard, but as above mentioned these data are important to understand the epigenetic control of genes that participate in leprosy immunophysiopathology. It is believed that future studies may reveal mechanisms to reactivate neural regeneration genes, in this case, the expression of piRNAs should also be modulated to avoid inhibiting regeneration.

### 2.4 circRNAs

CirRNAs are ncRNAs produced from an alternative splicing of the precursor mRNA (pre-mRNA), performed in the spliceosome, called back-splicing, in which the 3′end of an exon connects to the 5′end of an upstream exon via a 3′, 5′-phosphodiester bond, forming a closed linker structure with a back-splicing junction site (Li et al., 2018b; Chen, 2020; Zhou et al., 2020).

Some circular RNAs play an important role in gene regulation, modulating transcription and splicing, as well as in pathophysiological processes, acting as a miRNA sponge, which further suppress transcription and lead to gene silencing, as well as interacting with proteins and acting as models for polypeptide synthesis (Ojha et al., 2018; Chen, 2020). In addition, CircRNAs are involved in innate immunity, cell proliferation and transformation and neuronal function, and their dysregulation is related to the malfunction of physiological processes, resulting in several pathological conditions, such as the development of cancer, neurodegenerative diseases, tuberculosis and other infectious diseases (Li et al., 2018b; Ojha et al., 2018; Chen, 2020), but its involvement in leprosy is still unclear.

One of the only studies that associated circRNAs with leprosy was the work carried out by Gao et al. (2022) where they developed a circRNA–miRNA–mRNA network using MLO-Y4 murine osteocyte-like cells treated with N-glycosylated MDP (N.g MDP) to elucidate bone remodeling activity in leprosy. In this study, 724 differentially expressed mRNAs and circRNAs were observed between samples treated with control and N.g MDP, with 579 upregulated genes and 145 downregulated genes in differentially expressed mRNAs and 309 upregulated and 415 downregulated circRNAs, in addition to 58 pairs of circRNA–miRNA–mRNA interaction (Gao et al., 2022).

## 3 Perspectives

Noncoding RNA are implicated in a wide variety of human diseases, including infectious diseases. Recently, the use of RNA technology against COVID-19 has caused a “boom” in the study of ncRNAs as transcription modulators. NcRNAs have also been identified as important novel regulators of infectious disease risk factors and cell functions and are thus important candidates to improve diagnostics assessment. Beyond their application in diagnostic, ncRNA can also be the targets or tools of novel therapeutic strategies.

In recent years we have seen the growing number of studies related to ncRNAs, however there are still few studies related to leprosy. There is also a knowledge gap regarding the role of host ncRNAs in the etiology, diagnosis and development of vaccines against neglected human diseases. The expression profile of ncRNAs is a key element explored in the development of diagnostic biomarkers with greater sensitivity and specificity and reliable prognoses in leprosy. There is a lot to improve our understanding of leprosy pathophysiology. Therefore, future work should be developed to provide critical information for the development of preventive and therapeutic proposals, using ncRNAs as biomarkers for this neglected disease. We provide an update on recent developments and perspectives for diagnostic use of ncRNAs in leprosy diseases and new therapeutic targets in different forms of leprosy infection.
